# The diversity of biological models for bio-inspired aerosol filters

**DOI:** 10.1098/rsif.2025.0221

**Published:** 2025-06-25

**Authors:** Leandra Hamann, Timothy Foat, Alexander Blanke

**Affiliations:** ^1^ Bonn Institute for Organismic Biology, Section 2, Animal Diversity, University of Bonn, Bonn, Germany; ^2^ Defence Science and Technology Laboratory, Salisbury, UK

**Keywords:** aerosol filtration, biomimetics, bio-inspiration, air pollution, nasal cavities, wind-pollination

## Abstract

Innovative filtration systems are essential to enhance air quality or improve aerosol sampling for analysis, while addressing challenges such as high energy consumption, clogging and inefficiencies in capturing a wide range of particle diameters. Bio-inspiration provides novel design strategies by translating natural particle separation mechanisms (PSMs) into more efficient, adaptive and sustainable filtration technologies. This review systematically identifies six organismic groups as biological models that use distinct particle capture mechanisms to retain airborne particles for nutrition, reproduction and protection. Filtration-based PSMs in air, such as insect spiracles, hornet silk caps and spider webs, employ dead-end filtration with varying mesh structures to either purify air or capture prey. Non-filtration PSMs, including nasal cavities and wind pollination, rely on passive aerodynamic mechanisms such as impaction, interception and settling for particle retention. Flow regime control is crucial for non-filtration PSMs, where structures like nasal turbinates and pine cone surfaces optimize local airflows. Adhesive mechanisms, found in spider webs and nasal mucus, improve particle attachment. By mapping these principles to aerosol filtration challenges—such as particle adhesion, flow optimization and efficient removal of submicrometre particles—this review identifies promising pathways for bio-inspired aerosol filters in environmental monitoring, industrial hygiene and public health.

## 1. Introduction

Anthropogenic activities such as burning fuel, industrial processes and abrasion, e.g. tyre abrasion, have been significant sources of atmospheric aerosols for at least 200 years [1]. These aerosols contribute to air pollution, particularly in urban areas and have profound environmental and health impacts [1,2]. Fine particulate matter (PM), a major component of air pollution, affects respiratory health causing asthma, chronic obstructive pulmonary disease (COPD) and lung cancer. Additionally, prolonged exposure contributes to cardiovascular conditions, mental disorders and developmental issues during pregnancy and childhood [3,4]. Long-term mortality rates can increase up to 26% for people living in areas with elevated PM levels [5]. The World Health Organization (WHO) estimates that air pollution is the second leading cause for non-communicable diseases after tobacco [6]. In particular, small PM with a diameter of 2.5 µm or less (PM2.5) can penetrate deep into the respiratory system and enter the bloodstream, carrying toxic substances like heavy metals, e.g. lead or organic compounds, such as polycyclic aromatic hydrocarbons (PAHs) [7,8]. Mitigation strategies to reduce air pollution and achieve good air quality standards include sustainable transportation with less fine dust emissions, solid waste management, access to clean household fuels and cooking stoves, increased use of renewable energies and energy efficiency, and reduction of agricultural emissions from livestock facilities [9].

While broad mitigation strategies aim to reduce emissions at the source, air filtration remains a crucial technology for removing harmful aerosols in local environments. Filtration technologies play a critical role in managing airborne particulates, serving two primary purposes: removing unwanted particles from air streams to purify the air (clarification) and collecting valuable particles (recovery), for example, for enabling aerosol sampling for analysis [10]. A common application of the former is the removal of by-products from industrial processes before the gas or air can be re-used or released into the environment [10]. Another application is filtration in building heating, ventilation and air conditioning (HVAC) systems, to improve air quality within the building [11]. A similar approach is often applied in hospitals where high-efficiency particulate air (HEPA) filters might be used, either installed in the HVAC system or in a standalone unit [12]. Air-cleaning filters are also used on a much smaller scale, such as the use of face masks to reduce the spread of disease from or to the wearer [13]. In addition to air purification, aerosol filtration is essential for sampling applications, such as monitoring occupational exposure to dust [14] and detecting pathogenic bioaerosols in medical and environmental settings [15]. However, highly efficient filters often require significant pressure drops, leading to high energy consumption. Additionally, filters operating in contaminated environments can become clogged, reducing performance and requiring frequent maintenance [10]. These limitations highlight the need for innovative filtration strategies.

In order to improve current filters and develop new technologies, a biomimetic approach was chosen to find and analyse biological systems that have adapted to natural atmospheric aerosols. Natural atmospheric aerosols originate from natural sources such as mineral dust, sea spray, volcanic activity or biological sources [5]. Bioaerosols describe biological material, such as pollen, fungal spores, bacteria, viruses and fragments from plants and animals [16]. These natural aerosols are an integral part of the atmosphere and have formed the environments that we live in. For example, particles can alter climate through scattering and absorbing solar and thermal radiation and act as cloud condensation nuclei [17]. Transportation of aerosols, in particular, the smaller size fraction, can happen across large scales. For example, atmospheric transport mechanisms transfer significant amounts of mineral dust between continents and influence soil fertility [18]. Similarly, the circulation of airborne microorganisms between regions leads to distribution in remote environments [19]. Other lineages, such as wind-pollinating plants, have adapted to atmospheric particle transport mechanisms. Pollen dispersed by pine trees can be found up to 2600 m up at the atmospheric boundary layer and attached to flowers hundreds of kilometres away [20]. Organisms have evolved a variety of adaptations to interact with airborne particles, from capturing pollen to filtering bioaerosols. However, while a systematic review exists on aquatic particle separation mechanisms (PSMs) and their biomimetic potential [21], a systematic screening of biological models (BMs) for aerosol filtration remains largely unexplored.

This study aims to identify biological organisms and structures that capture airborne particles and evaluate their potential for bio-inspired filtration. Using a systematic literature review, we extract key functional traits, working principles and feasibility aspects for technical applications in aerosol filtration.

## 2. Screening approach for biological models

This review covers the first phase of the problem-driven (‘technology pull’) biomimetic process, specifically the transition from technology to biology [22]. The transition starts with analysing the technical problem, abstracting it into a functional principle and identifying potential BMs that address the problem. BMs refer to species or higher taxonomic groups that exhibit functional principles relevant to the initial problem. While this review focuses on the initial phase, the second phase involves selecting, analysing and abstracting a BM before translating it back into a technical application [22]. This phase, however, falls outside the scope of this review.

Particle separation from air is the initial technical problem to start the problem-driven biomimetic working process. Particle separation involves separating one or more distinct phases from another by using physical differences in the phases. Common separation techniques include evaporation, sedimentation, centrifugation and, most importantly, filtration [10]. In a filter, a filter medium, which is often fibrous or porous, is held across a fluid to retain particles on its surface (surface filtration) or inside its pores (depth filtration) [10]. Industrial filters must be efficient, allow high airflow and be robust, cost-effective and easy to maintain [10]. Some filters are single use (e.g. face masks or glass-fibre filters for air sampling) so do not need to be cleaned or repaired.

**Figure 1.**
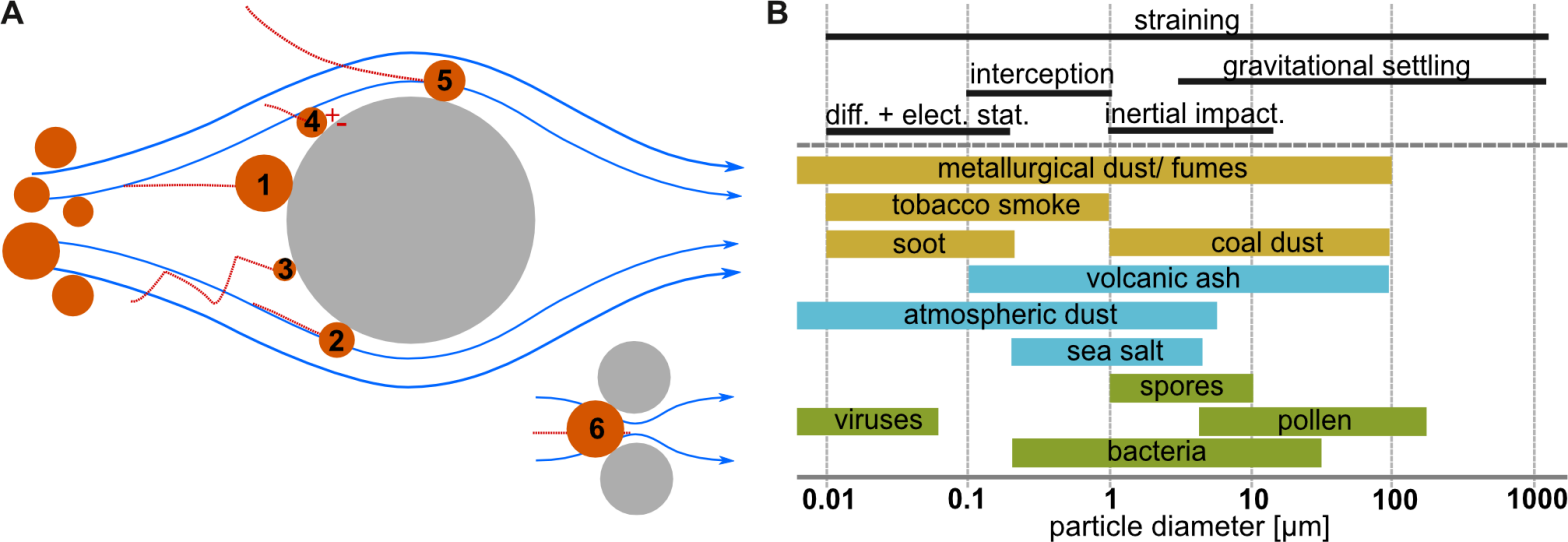
(A) Schematic drawings of PSM according to the AFT with (1) inertial impaction, (2) direct interception, (3) diffusion, (4) electrostatic attraction, (5) gravitational settling and additionally (6) straining. (B) The particle diameter is one factor that determines the dominating PSM. The AFT-relevant sizes include organic (green), natural inorganic (blue) and anthropogenic (yellow) particles. Modified from [5,10,23].

The aerosol filtration theory (AFT) provides a theoretical framework for describing PSM in air using a filtration medium. It is true for a single particle encountering a single fibre of a separation medium under laminar flow conditions [10] and covers five PSMs: inertial impaction, interception, diffusion, electrostatic attraction and gravitational settling (figure 1A). Depending on airflow velocity, collection fibre diameter and particle characteristics, such as size, shape and material, one of the five PSMs will be dominant [10,24] (figure 1B). Inertial impaction is dominant for larger particles (greater than 1 µm diameter), which deviate from airflow streamlines due to their inertia and collide with filter fibres. Interception affects medium-sized particles (0.1−1 µm diameter) that follow the airflow but make contact with fibres due to their size and proximity. Diffusion is relevant for smaller particles (less than 0.1 µm diameter), which undergo Brownian motion and randomly collide with fibres. Electrostatic attraction occurs when oppositely charged particles and fibres interact, making it effective for capturing particles with a diameter typically less than 1 µm. Gravitational settling mainly affects larger particles.

Another separation mechanism is straining, which is typically used in screening or filtration [10]. Particles that are larger than the distance between two neighbouring fibres (mesh size), are physically blocked and retained. Each of the AFT mechanisms, including straining can work independently or in combination, depending on the characteristics of the particles and the separation medium. The overall efficiency of an aerosol separation technology is determined by the interplay of these mechanisms to retain particles. AFT is equally applicable to organic, inorganic and anthropogenic particles (figure 1B) in technical and biological processes [25]. Technical processes are often designed to utilize and optimize these mechanisms for specific applications. In nature, evolutionary selection processes have led to PSM in BMs that are optimized for their biological function and environmental fitness.

AFT served as the foundation for identifying BMs, guiding our keyword selection for the literature search. The literature search aimed to find a large number of BMs, because the greater the number of BMs that can be identified for the given technical problem, the likelier a suitable match is found to ultimately design a bio-inspired solution [26]. As there was no specific application defined, this remained open at first. Additionally, there is currently no definition of organisms that use PSM in air, as there is for suspension feeders in water [21]. Therefore, the literature search was carried out very broadly, including separation and filtration mechanisms, and it combined search approaches from the biomimetic working process [27] and guidelines for systematic reviews [28].

We conducted three searches on Web of Science: one search query for bio-inspiration (BI-query), another one for biological studies (B-query) and a third one for AFT (AFT-query). These queries were refined over multiple iterations, incorporating relevant keywords for each category. An initial set of keywords and their synonyms were collected for filter (‘filter OR filtration OR retain OR particle retention OR particle separat*’), air (‘air OR aerosol’) and aerosol filtration theory (‘aerosol filtration theory’) for the AFT-query. To set the technical terms in the context of biology, the initial keywords were combined with keywords from biomimetics (‘bio-inspir* OR biomim*’) to formulate the BI-query and with biology (‘organism* OR animal OR species OR biolog*’) for the B-query. This led to a total of three search queries for the BM search (electronic supplementary material, table S1-1).The search queries were then fed to the Web of Science core collection, all results were exported in Bibtex format and organized in Mendeley Desktop (version 1.19.8) for reading. After the first round of results, the search queries were optimized based on additional keywords which were found when checking the first round of results. For example, ‘gas*’ was added as additional synonyms for ‘air’ and ‘aerosol’ because this is a term often used in this research field (electronic supplementary material, table S1-1). For queries yielding over 1000 publications, the Bibtex file was analysed in the R programming environment (R package bibliometrix [29]) to identify the 300 most common words with more than three letters that could be excluded in the search query to reduce the number of results (see third iteration in table 1). For comparison, we also searched the established database AskNature (https://asknature.org/ [30]) for ‘filter’, ‘filter air’ and ‘aerosol’. None of the search results covered particle separation in air.

**Table 1.** Number of publications for each biological model (BM), related to the first and second step of the literature search. The search queries were focused on biology (B), bio-inspiration (BI) and the aerosol filtration theory (AFT). The total number of publications is the sum of publications for all BMs.

		first step			second step		
		B-query	BI-query	AFT-query	new	double	total
1	phytoremediation	68	0	0	NA	NA	68
2	nasal cavities	37	1	0	60	6	91
3	wind pollination	1	0	0	6	0	7
4	insect spiracles	1	0	0	0	0	1
5	hornet silk	1	0	0	0	0	1
6	spider webs	0	2	0	2	0	4
	total	108	3	0	68	6	172

Studies were considered relevant if they investigated bio-inspired air filters or BMs capturing particles greater than 10 nm, the lower limit of AFT. Biological mechanisms such as odour and pheromone detection were excluded from this review as these concern detection of chemical components below 10 nm size. Examples are odour detection in the ethmoidal region in nasal cavities [31] and chemo-sensation in insects, e.g. pheromone detection by antennae [32,33]. Studies were also excluded if they were water related (water filters or suspension feeders), covered the development or application of solely manufactured filters and others (monitoring methods, contamination and medical reports, etc.). We excluded studies that were about bio-inspired manufacturing technologies and designs that are not related to a PSM in a BM, e.g. bio-inspired fibres [34] or surface patterning of filter media based on the water-repellent lotus leaves [35]. These optimizations might be interesting at a later design stage, but initially we focused on biological models with a full-featured PSM in nature. Finally, we excluded publications that primarily dealt with the physiology and medical effects of aerosol/particle deposition and retention. The remaining publications were grouped together based on the biological models for the three search queries. The fraction of the total number of publications that were considered relevant was calculated if they did not exceed 3000 publications (electronic supplementary material, table S1-1). That share of relevant publications is a first approximation of the search efficiency to find information on BMs of each search query.

In a second step, all remaining publications were screened to identify new keywords describing each BM. The biological keywords were combined with ‘particle’ and verbs to describe the desired process (‘retain’ OR ‘separate’ OR ‘attach’, electronic supplementary material, table S1-1). This aimed to broaden the search for publications focusing on the functional part of PSMs in each BM [27]. The results were again screened for relevant literature and the search efficiency was calculated if the results were greater than 3000 publications.

In total, six BMs were identified (table 1). The B-query identified 108 relevant publications for five of the six BMs. Based on the total publication for this search query, the relevant publications have a share of 3.92% (electronic supplementary material, table S1-1). The BI-query only led to the identification of two BMs with a total of three relevant publications. Although the total number of results could be slightly reduced with the optimization of the search query, the share of relevant publications still remains relatively low with 2.07% (electronic supplementary material, table S1-1). The most relevant publications were related to phytoremediation followed by respiratory tracts and spider webs with 68, 38 and 2 publications, respectively. Three BMs, namely pollination, insect stigmata and hornet silk caps were only indicated by one publication, respectively. The AFT-query yielded no relevant publications.

The second step identified 62 additional relevant biology-related publications (table 1). This does not include any new publications on phytoremediation, because the results were too many and the number of relevant publications from the first step were considered sufficient to describe the general principle. For all other BMs, the share of relevant publications from the total results increased compared with the first step due to the more focused search queries. The number of relevant publications that came up in the second step that had already been found in the first step (‘double’ in table 1) was surprisingly low. This indicates that a change from a broad approach to search queries targeted towards the identified BMs is an important step in the search strategy. Of a total of 172 relevant publications, only three publications were related to bio-inspiration. A selection of the relevant publications complemented by secondary and additional literature was used to describe the PSMs in each BM in §3. To describe elemental characteristics and functions [36] and compare the BMs among each other and to engineered filters, a set of parameters previously developed for suspension feeders by Hamann and Blanke ([21] and their supplement for parameter definition) was used to organize the data.

## 3. Biological models

### 3.1. Phytoremediation and particle deposition on plants

**Figure 2.**
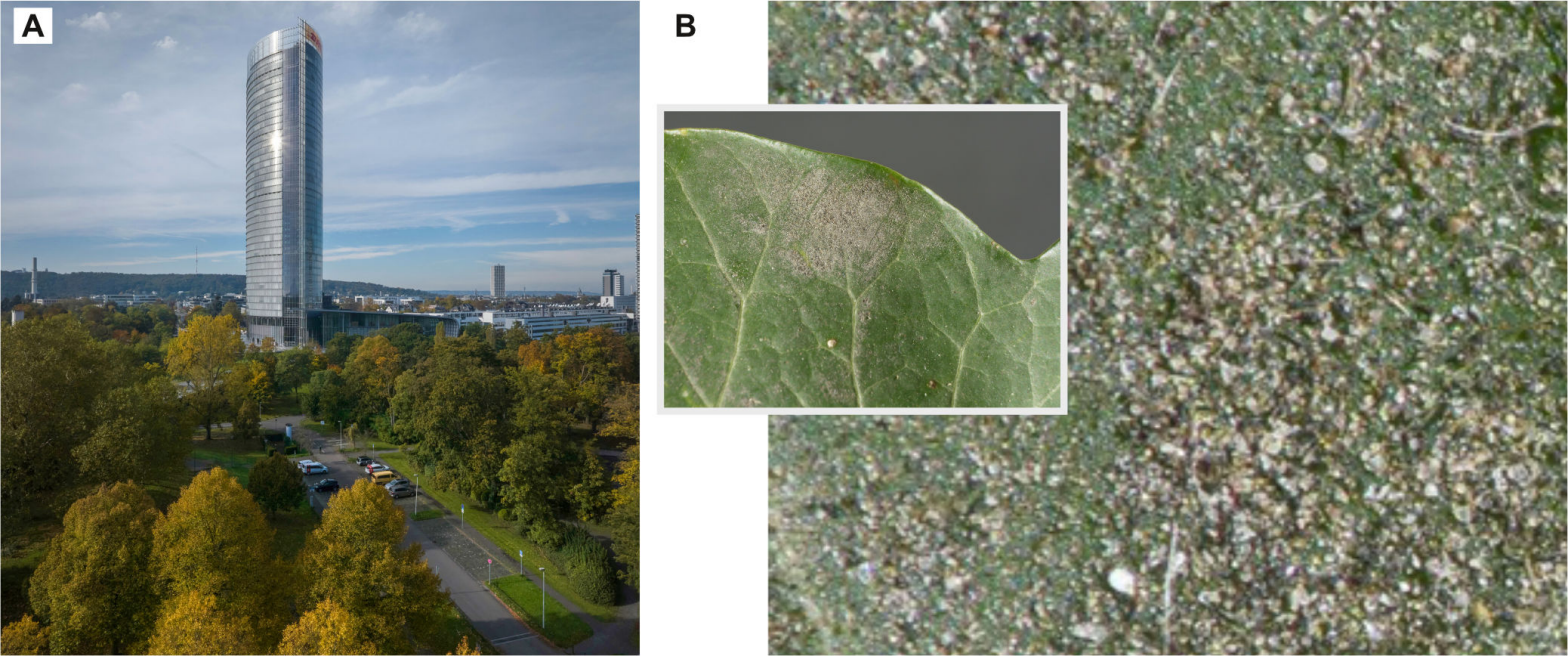
Phytoremediation describes the air-cleaning effects of plants through particle deposition. (A) Urban vegetation in the city of Bonn, Germany (photo: Volker Lannert/Uni Bonn). (B) Aerosol particles accumulated on a leave collected close to a main road in Cologne, Germany (photo: Jens Hamann).

Phytoremediation refers to the use of plants to remove contaminants from soil, water and air. Plants are an established measure to reduce PM (less than 10 µm) in urban areas (figure 2) [37], a mechanism also known as ‘phytofiltration’ [38]. Particles can be washed out of the atmosphere during precipitation events or deposited on leaves (figure 2) and bark due to sedimentation, inertial impaction, direct interception, diffusion and even electrostatic attraction between the particles and plant surfaces [38–40]. Once deposited, particles can be dislodged by wind, washed away by rain or removed when deciduous trees shed their leaves [41–43]. Ultrafine particles may also enter the plant through the stomata, where heavy metals, PAHs and other toxic components of the particles can disturb the physiology of the plant [37]. Phytoremediation does not imply an inherent cleaning mechanism exhibited by the plant itself and particle deposition is rather a by-product of the plant’s exposure to an environment than a functional adaptation. However, certain plant characteristics can enhance particle deposition, making them still of interest for bio-inspired filters.

Particle retention varies among plant species and is influenced by traits such as tree size, crown shape, leaf morphology, total leaf area and seasonal growth patterns [41,43]. Evergreen trees are generally more effective at capturing particles than deciduous trees, as they retain foliage year-round, providing continuous surface area for deposition [38]. Large conifers remove more particles than broadleaf deciduous and broadleaf evergreen trees over the course of a year [41]. Relevant traits of the leaf macrostructure are shape, size, leaf blade margin and leaf petiole [40]. For example, leaves with a longer petiole move more in the wind and prevent particle deposition [40] and narrow conifer needles are more efficient in particle capture compared with flat leaves due to their shape, surface structures and surface waxes [41]. Leaf microstructures such as grooves, protrusions and trichomes enhance particle retention by increasing surface roughness and reducing resuspension [37,42]. Additionally, higher cuticle wax content leads to high particle accumulation [37,40]. Particle retention increases also with the bark surface roughness that is formed by cracks, exfoliation and lenticels [38,42]. Barks generally showed a higher amount of retained PM per unit area than the leaves of the same trees of five species [42]. At the whole-plant scale, the total leaf area is around 10 times larger than the total bark area, which increases the chances of particles encountering a leaf [42].

Wind tunnel experiments demonstrated that particle capture efficiency depends on the particle’s Stokes number, which describes the balance between inertial and viscous forces. Higher Stokes numbers correspond to greater particle capture efficiency, although this relationship varies among plant species [39]. For example, a greater wind velocity increases the particle’s inertia and leads to more effective deposition, but flat leaf surfaces can result in particles bouncing off [39].

### 3.2. Respiratory tracts and nasal cavities

**Figure 3.**
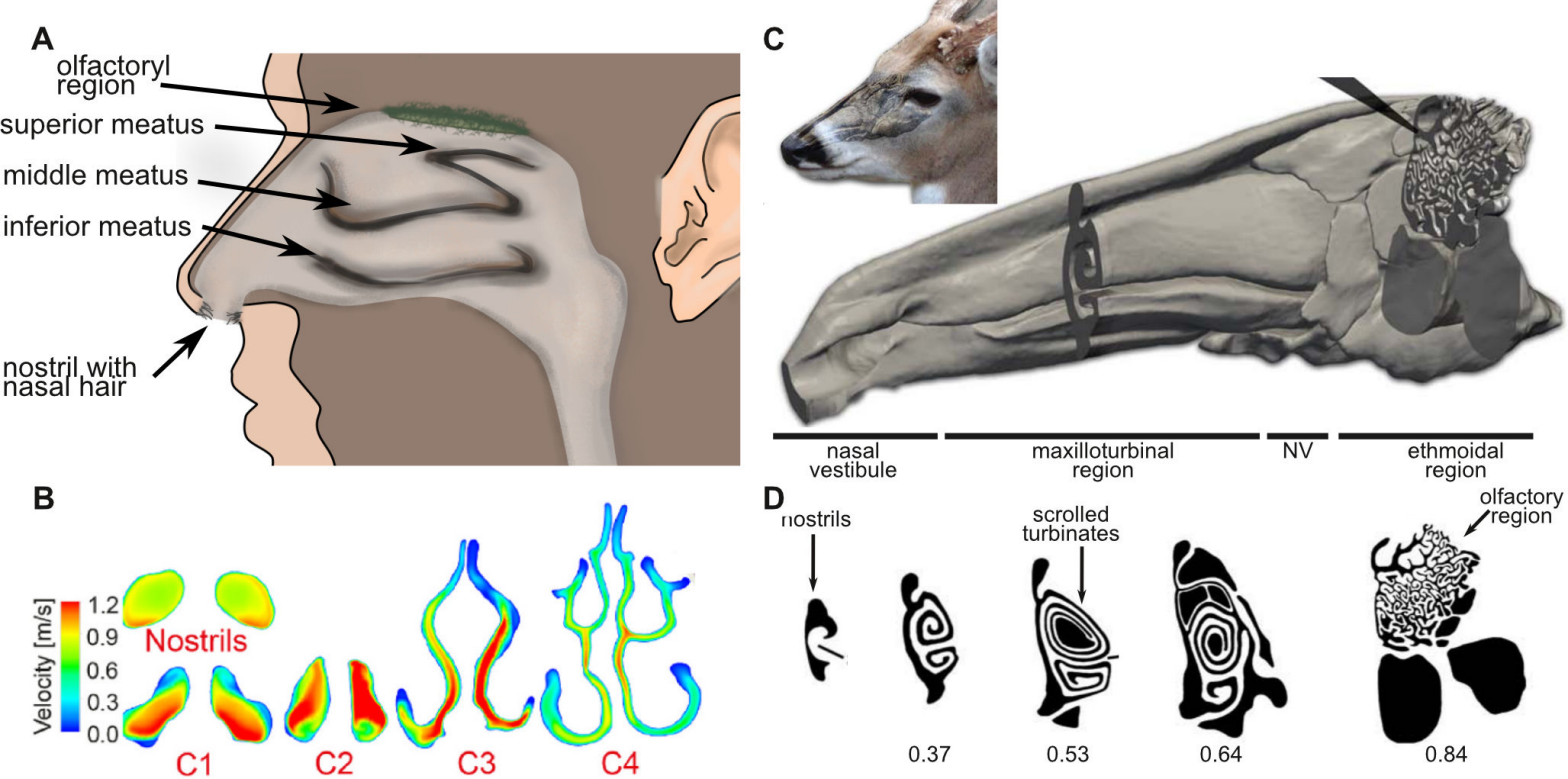
Nasal cavity morphology in humans and the white-tailed deer *Odocoileus virginianus*. (A) Anatomy of the human nostrils and nasal cavities. (B) Cross-sections through the folded turbinates and modelled flow velocities indicated by colours. Adapted from Yan *et al*. [47], with permission from Elsevier [47]. (C) Position of the nasal cavities in the white-tailed deer and computed tomography scans show four regions: nasal vestibule, maxilloturbinal region, nasomaxillary region and ethmoidal region. (D) Cross-sections through the different regions along the longitudinal axis (black = space filled with air, white = animal tissue). C and D are adapted from Ranslow *et al*. [44], with permission from John Wiley and Son.

The respiratory tract facilitates gas exchange between the body and the environment. The upper respiratory tract, which includes the nose, nasal cavities and pharynx, serve respiratory air conditioning and thermoregulation, olfaction and filtration [44,45]. The lower respiratory tracts consist of the trachea, bronchi, bronchiole and alveoli in the lungs where oxygen and carbon dioxide are exchanged with the blood. About 80% of the inhaled PM is retained in the upper respiratory tract [46]. Therefore, we will focus on this area and specifically the nasal cavities. The nasal cavity is divided into two parts, the external nostrils and internal nasal cavities (figure 3). Despite differences in morphology, the basic structure of the nasal cavity is conserved across mammals, reptiles and birds. It always consists of the bilaterally paired nasal organs with two nostrils (or nares), the two choanae connecting to the mouth, an olfactory chamber and the nasopharynx connecting to the lungs. However, nasal morphology varies among species. For example, sand lizards possess U-shaped nasal cavities, while white-tailed deer exhibit highly scrolled turbinates [45].

In mammals, inhaled air moves through four nasal regions: the nasal vestibule (NV), maxilloturbinal region (MR), nasomaxillary region (NM) and ethmoidal region (ET). Particle deposition occurs throughout the nasal cavity (figure 3). However, only a small fraction (approx. 1%) of inspiratory airflow reaches the ET, which primarily functions in olfaction [44,45]. Particles larger than 10 nm, the primary focus of this review, predominantly deposit in the NV and MR. The NV is lined with stratified squamous epithelium, similar to the skin epidermis, whereas the MR is lined with respiratory ciliated epithelium that contains the mucociliary system [44]. The mucociliary system removes inhaled particles by trapping them in mucus secreted by goblet cells and submucosal glands. Ciliary motion then transports these trapped particles out of the airways [48]. Larger particles may also be expelled through coughing [49].

The human nasal cavities are well studied and it was found that several morphological traits influence airflow and particle deposition: change in direction, wall roughness, the presence of nasal hair and the position of the nose within the face. The human nasal cavity features three approximately 90° bends: (i) the narrow nasal entry with a stagnation point, (ii) the top of the meatus wall or middle turbinate, and (iii) the region near the nasopharynx [47,50]. The fastest flow velocities are at the entrance of the nasal cavities with up to 2.4 m s^−1^ during tidal breathing [47]. The fast flow in combination with the first bend in the nose capture most 10 and 20 µm particles in this part of the nose [47,50]. Inertial impaction is the dominant mechanism of deposition, especially for particles greater than 5 µm at flow rates 7.5–15 l min^−1^ [51] and up to 20 l min^−1^ [50]. For particles less than 5 µm, the diffusion mechanism is dominant. Airflow inside the nasal cavities transitions to laminar flow and remains laminar, even at mean flow rates of 24 l min^−1^ [50]. Investigation of different breathing patterns in computer models shows that particle deposition is higher at elevated constant flow rates rather than during fast inhalation and exhalation. Additionally, including a wall roughness of 0.2 mm that mimics mucus in the computer models increased particle deposition significantly, compared with smooth walls. This effect was especially high for larger particles [50]. Those effects are minor for small particles (less than 2 µm diameter). Nasal hair, which grows at the nostril’s entrance, probably contributes to particle retention, although studies are limited [52]. Computational fluid dynamics (CFD) simulations with varying hair diameters (40, 95 and 120 µm) and 3 mm hair length demonstrated that nasal hair increases particle deposition near the nostrils and improves airflow distribution. Thicker hair enhances retention, primarily through inertial impaction and interception [52]. The protruded nose within the face allows sampling of around 180° in the breathing zone [53]. In CFD experiments, the flow patterns change when a face is modelled in addition to nasal cavities. When a face was modelled, additional vortices formed inside the nasal cavities that transported the particles deeper inside the nasal cavities. This effect was strongest for particles with 10 µm diameter and less strong for particles with 2.5 and 20 µm diameter [53]. Other factors, not related to morphology, can also influence particle deposition in the human nasal cavities, e.g. wind speed and direction, particle shape and surface charges, head position, motion, body size and facial hair [54].

Nasal cavity morphology is more complex in many non-human animals. For example, the turbinates in humans are larger compared with dogs, pigs and rabbits based on radius of curvature, gap thickness and tortuosity [31]. Tortuosity is an anatomical descriptor to quantify and compare the turbinate morphology across animals based on the ratio of curved paths in an anatomical tract over the linear spatial distance, between two points [31]. The gross morphology can also be categorized into four types: folded, single scroll, double scroll and branching [31,45]. In white-tailed deer, turbinates increase surface area and maintain laminar flow while supporting high volume flow rates of 14.1 l min^−1^ at 0.3 Hz breathing frequency [44]. Similar to humans, the airflow in other animals is parallel to turbinate’s surfaces. This cross-flow in combination with reduced flow velocities, creates laminar flow conditions with Reynolds numbers (Re) of less than 500 in the elongated MR region of white-tailed deer and allows the particles to deposit on the nasal cavity walls through direct impaction and gravitational settling [31,53,55]. Further descriptions of mammalian nasal cavity morphology can be found for example for the mole rat nose in Zuri *et al.* [56], the bat noses in Id and Smotherman [57], the mouse and rat nose in Wu *et al.* [58], and domestic and wild animals in Xi *et al.* [45] and Yuk *et al.* [31]. The sandfish lizard is an example for adaptation to an extreme particle load during breathing as it buries itself in the desert sand to evade the heat [49]. The sandfish lizard’s nasal cavities show an elongated U-shaped tube in which the inhalant airflow is abruptly slowed down so that sand particles can sediment and impact with the mucus-covered epithelium [49,55].

### 3.3. Wind-pollination and pollen capture

**Figure 4.**
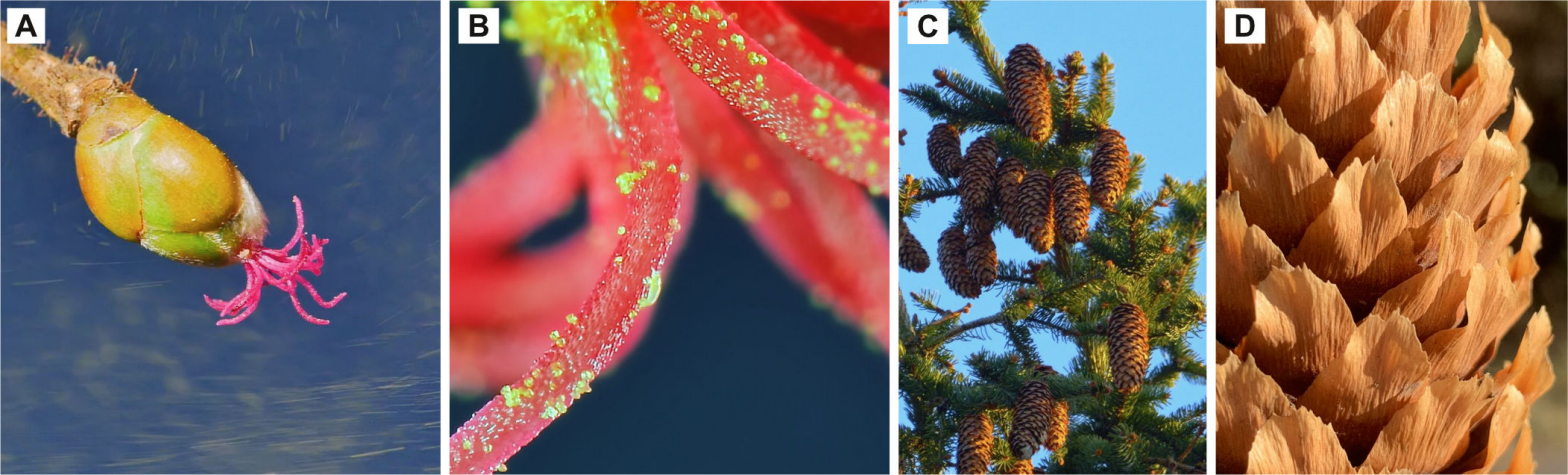
Wind pollination in the common hazel *Corylus avellana*. (A) Pollen is flying around the small female flower and (B) captured on the exposed stigma. (C) Pine cones hanging from a tree with (D) individual cone scales. (photos: Jens Hamann)

Pollination is the transfer of pollen from male anthers to female stigmas, enabling fertilization and seed production (figure 4). Pollination occurs through two main strategies: abiotic pollination, where wind (anemophily) or water (hydrophily) disperse pollen, and biotic pollination, where animals such as insects (entomophily) or vertebrates (zoophily) transfer pollen [59]. Wind pollination consists of three physical processes: pollen release, dispersal and capture [60]. Wind-pollinating species are found in several groups within the seed-producing plants (gymnosperms) and in around 20% of families in flowering plants (angiosperms) [59,61].

In wind pollination, wind provides a random and relatively inefficient mode of pollen dispersal in which pollen is carried along the airstream as individual grains [62,63]. To compensate for inefficient pollen dispersal, wind-pollinated plants produce large quantities of pollen, synchronize flowering periods and develop specialized pollen morphologies [60,64]. Additionally, they maximize pollen capture by producing numerous small, widely distributed female flowers that increase exposure to air currents [63]. Often, the individual female flowers have reduced perianth parts to expose the female receptive surfaces to the wind that carry pollen [62,63]. Whether a pollen grain impacts on the stigma depends on wind speed, stigma size, pollen diameter and pollen density [60,62,63]. Pollen grain size varies from 5 to 250 µm [60,62]. Larger, denser grains are more likely to be captured by inertial impaction, whereas smaller grains tend to follow airflow streamlines and bypass the stigma [63]. Most wind-pollinated species produce pollen grains between 20 and 40 µm, balancing long-distance dispersal with the ability to deviate from airflow and reach the stigma [60]. Due to their size, pollen grains have low gravitational settling velocities, and, additionally, wind speeds, e.g. in forests, can reach 1−10 m s^−1^ thereby enhancing pollen transport [60]. Higher wind speeds improve pollen capture by increasing encounter rates and reducing boundary layer thickness around the stigma [60,62]. Larger stigmata generate thicker boundary layers, pushing airflow (and pollen) away [63]. In contrast, thinner boundary layers increase the likelihood of direct interception [10].

Pollen capture efficiency increases with surface area and fine structural elements [60,63]. Feathery stigmata in grasses exemplify this adaptation, maximizing pollen interception [60]. When the spikelets of grasses move in the wind, pollen grains encounter the upwind and downwind side and are captured by the stigmata through inertial impaction and sedimentation [65]. The grass motion induced by wind (collector motion) can increase capture efficiency by up to 400%, especially at low Reynolds numbers when flow is laminar [66]. Additionally, electrostatic attraction of a positive=charged pollen and a negative=charged stigma may also play a role in particle capture [62]. Pine cones optimize pollen capture through high surface area and specialized cone scale morphology (figure 4C,D), which alters local airflow to direct pollen near the ovules [67]. Pollen deposition in pine cones is also influenced by cone orientation. A pine cone tilted by 45° angle from its longitudinal axis with the cone tip pointing downstream maximizes capture efficiency [64].

### 3.4. Spiracle filtration in the arthropod gas exchange

**Figure 5.**
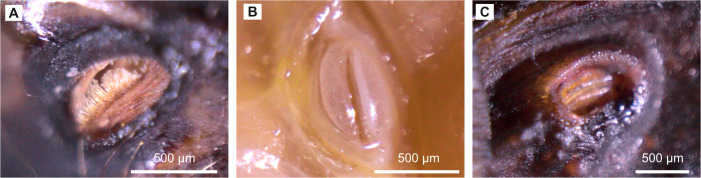
Spiracle openings in various insects. (A) Spiracle with filter setae on the thorax of the dragonfly *Aeshna cyanea*. (B) Narrow opening type spiracle on the thorax of the migratory locust *Locusta migratoria*. (C) Wide opening type spiracle on the abdomen of a phasmid *Eurycantha calcarata*. (photos: Leandra Hamann)

Spiracles are the external openings of the tracheal system, facilitating gas exchange in insects, myriapods, oniscoidean isopods, Onychophora and various arachnid groups [68–72]. The tracheal system is the primary mode of gas exchange in insects and, due to their large diversity, is the most widespread respiratory system among animals. The spiracle opening can be wide or narrow and many insect lineages can also actively close their spiracles to regulate gas exchange, alter airflow direction or prevent water ingress during diving [72–74]. To protect the inner tracheae from large particles and dust, several species have evolved varying morphologies at the spiracles ranging from cuticular setae (figure 5A) and hairs, to porous plates or reticulate structures [72,75]. To date, the filtering capacity of spiracles has not been extensively studied. Wagner *et al.* [76] examined spiracle scaling in beetles of various sizes and found that spiracle openings range from approximately 0.3 to 3.5 mm, limiting the size of particles that can enter. Larger Cerambycidae (Coleoptera) such as *Petrognatha gigas* show giant forward-facing spiracles and huge thoracic primary tracheae to achieve high volume flows during flight by using a form of ‘ram ventilation’ [77]. To get rid of particles blocking the spiracles insects can change flow directions so that air is pressed out of certain spiracles [78,79].

### 3.5. Silk cap filtration in hornet brood combs

**Figure 6.**
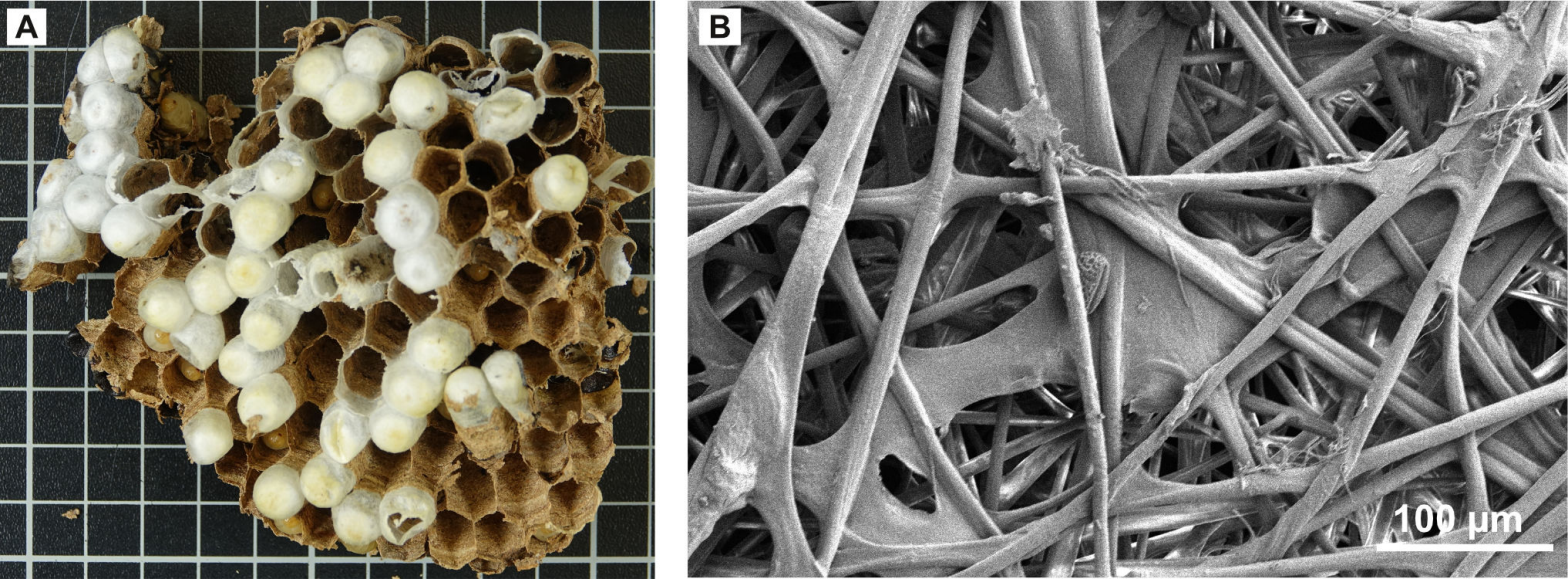
(A) Silk caps on brood combs of the Asian hornet *Vespa velutina*. (B) Scanning electron microscope image of silk strand layers in the silk caps. (photos: Leandra Hamann)

The order of Hymenoptera includes 144 000 insect species, including bees, ants and hornets. Silk production has evolved independently at least six times in Hymenoptera, primarily for constructing cocoons and nests [80]. To date, only one study has shown cocoons might also act as filters that achieve stable environmental conditions (29°C; greater than 90% relative humidity) to ensure optimal metamorphosis of larvae to adults during metamorphosis [81]. Apart from the climatic conditions, the interior of the comb cells also needs to be free of dust particles and pathogenic microorganisms [81]. For the oriental hornet *Vespa orientalis* and the Asian hornet *Vespa velutina,* this is achieved by the silk caps on the comb cells (figure 6A). These silk caps consist of multi-layered, cross-linked fibres with a diameter of 10−20 µm interwoven with silk plates (figure 6B). The silk cap is spun by the larvae and seems to follow a certain spinning procedure, which is different to the silk spinning in bees that involves random head movements [80]. Laboratory tests showed that silk caps retained greater than 99.9% of 1−30 µm particles from both liquid and gaseous suspensions [81]. Moreover, the absence of microorganisms in comb cells suggests that silk caps effectively prevent contamination. It was also found that the silk has hydrophobic characteristics, which led to an easy removal of accumulated particles (‘cake’) on the filter medium’s surface. After cake removal through flow reversal, the initial flow rate of 0.08 l min^−1^ achieved by a 2 bar inlet pressure in the experiments was restored, i.e. the filter was clean again [81]. However, as there is no driving force in the hornet nest pressing the air through the silk caps, this might not reflect natural nest conditions. After metamorphosis, adult hornets chew their way through the silk caps. Thus, silk caps function as single-use filters without the need for a self-cleaning mechanism.

### 3.6. Prey capture with spider webs

**Figure 7.**
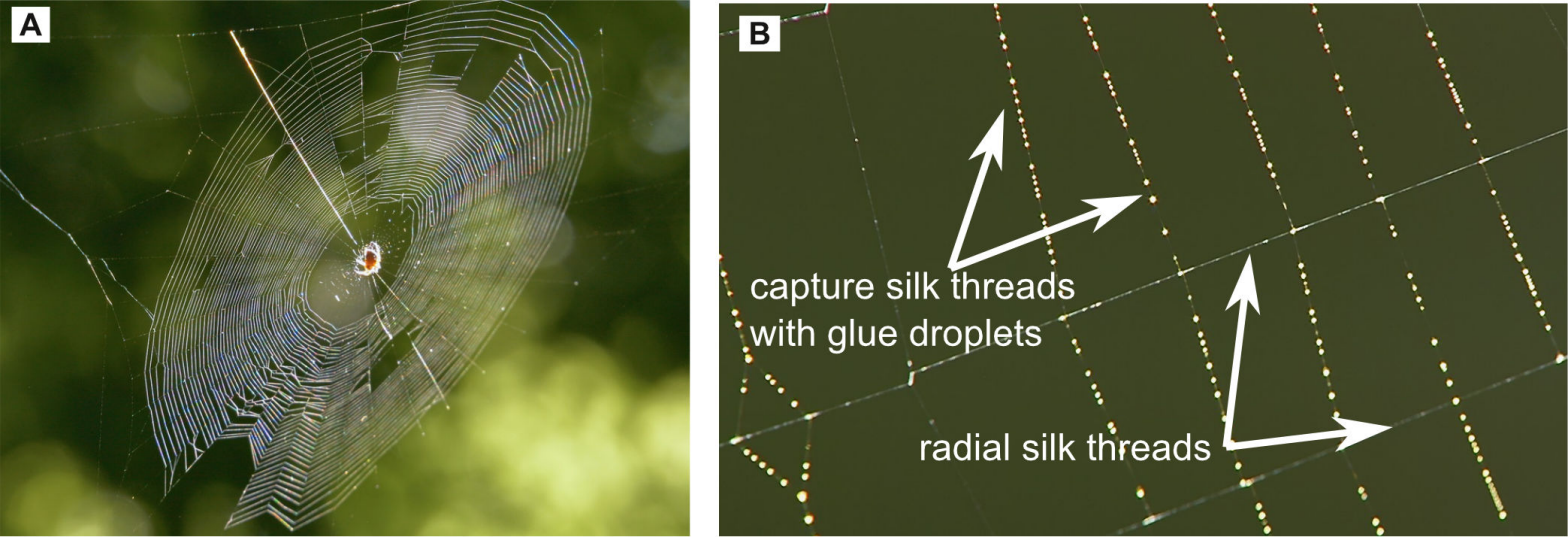
(A) Spiral orb web of a European garden spider (*Araneus diadematus*). (B) Capture silk with glue droplets and radial silk without glue droplets. (photos: Jens Hamann)

Spider webs efficiently trap organisms of their own size or larger, thanks to their remarkable material properties, particularly their strong adhesion [82–84]. Among various spider web designs, the two-dimensional, point-symmetrical orb web is particularly well-studied for its effectiveness in capturing flying insects (figure 7A). The orb web of the European garden spider (*Araneus diadematus*) spans approximately 230 cm² [85] and comprises around 27 radial capture threads and a spiralling thread forming 6–10 radii [86]. In *Araneus diadematus,* silk threads measure 1−2 µm in diameter, while in other species, diameters range from 300 nm [87] to 4.4–6.2 µm [88]. Mesh size varies among species, balancing prey interception, stopping efficiency, retention and aerodynamic factors [86,89]. Small prey can be captured with a single silk thread, whereas larger prey may break through, making smaller mesh sizes more effective for retention [86]. However, a larger web with denser meshes increases the drag induced by wind [89]. For a new net, the silk is spun from a coaxial spinneret on the posterior side of the spiders and varies in properties depending on the specific function of the spinneret [83,87]. A single capture silk fibre is a compound composed of inner paired axial fibres and outer regularly distributed glue droplets [82,83] (figure 7B). The inner fibres have high strength and extensibility to absorb kinetic energy of flying insects, while the outer glue droplets contain glycoproteins and hygroscopic low-molecular-mass compounds to stick to the prey [83,87].

## 4. Discussion and biomimetic potential

PSMs evolved in six different organism groups according to our systematic review. While phytoremediation is rather a by-product in which the plants act as vectors for a particle transfer from air to leaf surface or soil without a benefit for the plant itself, the other five PSMs evolved under selective pressure and they serve nutrition, reproduction and protection of body functions. These functions are also found in PSMs in water. For example, wind pollination corresponds to the external fertilization of spawning corals [90] and protection of respiratory organs such as lungs and the tracheal system is similar to the buccal tentacles in ascidians that protect their gills and internal structures [91]. Prey capture as seen in spider webs corresponds to suspension feeding mechanisms [21]. However, in contrast to PSMs in air, the number and diversity of PSMs in water are much higher. Just suspension feeding mechanisms in water alone account for at least 35 different PSMs [21]. We assume that the different fluid characteristics allow higher particle mass concentrations in water than in air, being a crucial factor for adaptations to suspended particles. Water is approximately 800 times denser and approximately 55 times more viscous than air. Based on Stoke’s Law, which describes the settling velocity of a small, spherical particle in laminar flow under the influence of gravity [62,92], a 10 µm particle with a density close to crustacean or insect tissue (approx. 1100 kg m^−3^, [93]) will fall for approximately 0.3 s mm⁻¹ in air and approximately 3 min mm⁻¹ in water. This allows for not only motile but also non-motile particles or organisms to remain suspended for a longer time. Particle mass concentrations in water are also higher, which is of relevance for organisms such as certain suspension feeders which only start feeding when a specific plankton concentration is exceeded (e.g. ram-feeding fishes [94]) while corals spawn all at once to increase the chances of egg and sperm encounter [95]. In air, larger particles have limited travelling time, e.g. for pollen distribution, or need to be motile to remain airborne, e.g. flying insects. Therefore, aerosol particles are smaller (less than 300 µm, figure 1B) than seston particles (less than 20 mm, [21]) to remain airborne and the mass per unit volume is much less in air than in water.

Because of the low number of biological models (BMs) and their different functions in air, convergent evolution of form–function patterns, as in suspension feeders, is less pronounced in the identified aerosol PSMs (table 2). One major difference is the presence of non-filtration mechanisms versus filtration mechanisms, the latter being defined as a filter medium held across a fluid to retain particles on its surface or inside its pores [10]. In three of the six identified PSMs, particles are retained through dead-end filtration in which the separation medium is perpendicular towards the flow. This includes insect spiracles, hornet silk caps and spider orb webs (table 2). Their separation media designs and mesh sizes differ with the particular purpose of the PSM. Hornet silk caps remove unwanted particles, which resembles a clarification process, whereas spider webs are built to increase chances for prey capture analogous to a recovery process [96]. Similar differences were found in the non-filtration processes, i.e. wind pollination (recovery) in contrast to particle removal in nasal cavities and insect spiracles which both purify air for gas exchange (clarification). Depending on whether it is a recovery or a clarification process, established performance metrics differ as well. Recovery is measured by the successful collection of the target particle relative to a total particle concentration, whereas clarification focuses on the purification of the fluid. Particle capture rates also largely differ in the BMs. For example, pollen capture efficiency in the grass *Alopecurus pratensis* ranged between 5 and 20% [97]. In the clarification of the air in nasal cavities, the purified air allows better gas exchange and reduces immune responses to potential harmful particles [98] with no further purpose for the retained particles.

**Table 2.** The biological traits of each biological model, i.e. phytoremediation (1), nasal cavities (2), wind pollination (3), spiracles (4), hornet silk caps (5) and spider webs, specifically orb webs (6), are clustered and presented for each parameter. The set of parameters was initially developed for suspension feeders [21], therefore, some cells are not applicable to aerosol PSM and remain open. The literature references are available in the electronic supplementary material, table S2-2.

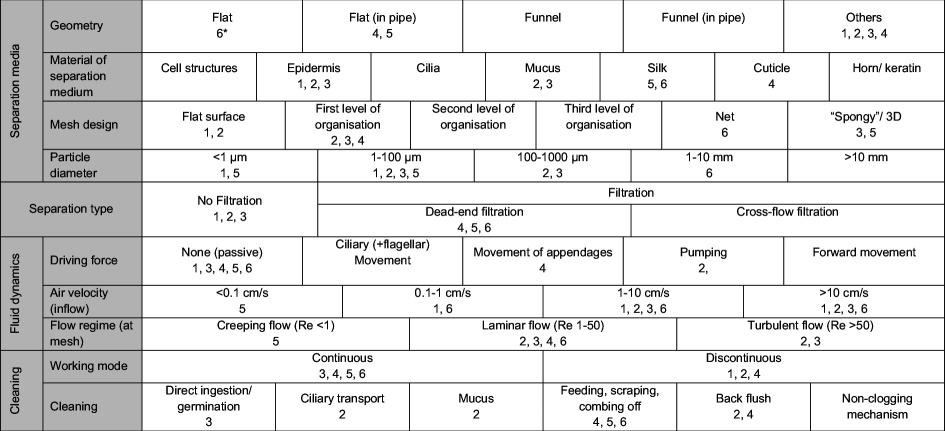

In the two BMs that do not use a filtration mechanism, i.e. nasal cavities and wind-pollination, particles are retained by flat or branched surfaces through a combination of aerosol PSMs (table 2). For example, interception, impaction and settling are the main mechanisms for particles larger than 10 µm in the nasal cavities of mammals (§3.2.). Especially for these BMs without a filter medium, flow regime control is important to induce particle encounter on the designated surfaces and create laminar to intermediate flow conditions. According to AFT, particles following the streamline will come closer to the fibre and the encounter rate will increase by 4.7 for Re 0.1 to 10 at intermediate Re, according to numerical models [99]. Female flowers are radially symmetric to perform omnidirectional sampling in wind with varying flow velocities and turbulent flow regimes. Thereby, the receptive organs channel local flows to induce particles deposition, as seen for pine cones [100]. In the nasal cavities, the total cross-sectional area increases while the channel (hydraulic) diameter decreases from the nostrils towards the nasopharynx. This decreases the flow velocity (conservation of mass) and reduces the ambient turbulence, and thereby increases particle deposition through gravitational settling on the folded turbinates [44]. The PSM in the nasal cavities has already been used in bio-inspired designs. May [101] described how his aerosol collection device, called a cascade impactor, was inspired by the human nose. This cascade impactor trapped larger particles while allowing smaller ones to pass through. It consisted of a curved pipe which was designed to provide a 10 μm cut-off. A later design describes ‘a device consisting of a straight glass tube ‘nostril’ impinging an air jet on to a microscope slide ‘turbinal’ then a curved glass tube ‘trachea,’ followed by a ‘lung’ consisting of a fine jet impaction stage backed by a filter’ [101]. May’s cascade impactor operated at 17.5 l min^−1^ [101]. Another example is the bio-inspired filter medium based on the inertial impaction of particles on the olfactory epithelium in the ethmoidal region in pigs and dogs. The bio-inspired filter showed low-pressure difference through particle deposition by inertial impaction induced by many changes in direction [31].

Inertial impaction has been a common method to separate particles from air since the 1860s [102]. A standard impactor consists of a nozzle that creates an airflow directed at a flat impaction plate onto which the particles impinge [102]. This fundamental design was adapted to various applications used for air quality monitoring, particle diameter classifications or medical aerosol inhalers [102–104]. As the primary purpose of impactors is to obtain representative samples and accurately measure the ambient particle distribution, optimization efforts have focused on improving pressure drop and volume flow rate in order to capture smaller particles, enabling real-time indication of collected particle mass and enhancing particle diameter characterization [102–104]. Even though this function deviates slightly from the clarification process during respiration, the structure of the nasal cavities could still be relevant for improving particle capture efficiency. The parallel walls formed by the folded turbinate inside the nasal cavity increase the deposition area, increase the chances of gravitational settling, decrease the distance to the nearest surface and, thereby, increase particle deposition (§3.2). This design may be of particular interest for use as a pre-filter for personal biological aerosol samplers. Samplers such as that described by Foat *et al.* [105], are typically required to exclude larger particles to collect a defined size fraction, e.g. respirable particles. A pre-filter inspired by the U-shaped nasal cavities of the sandfish lizard could be specifically applied to environments with high-density particles and high particle concentrations [55]. Additionally, nostril and pine cone morphology could be used to improve omnidirectional inlets for aerosol samplers or filters, such as the bell-shaped inlet [106], to reduce turbulence and set local flow regimes to optimize flow rates. First attempts in that direction were made based on the nasal cavities, for example, in a bio-inspired smell sensor that mimics the human nasal cavities [107], a three-dimensional-printed dog nose to improve commercial trace vapour detectors [108] and a spiral geometry inspired by the reindeer nose for catalytic micro-combustors [109].

Spiracle filtration, silk caps and spider webs show a more typical filter media design. In all three, the filter medium consists of fibres or fibre-like structures that form meshes or pores. In these BMs, the material has to withstand flow while ensuring particle capture. The mechanical properties of spider silk have long been of interest for engineers to be used in water collection, cell cultivation, drug delivery, anti-adhesion coating, adhesive glue and adhesive tape [83]. The usage for particle separation and filtration is less common but a few studies show the potential for the use of natural and artificial spider silk. For example, to capture micrometre-sized particles in bioaerosol survivability experiments, the natural thread from orb web spiders, such as *Araneus diadematus* and *Zygiella x-notata* was wound around a metal frame to create a mesh of fibres [110]. The aerosol particles stick to the fibres and the fibres’ small diameter allows the trapped particles to be exposed to the environmental conditions of interest, e.g. humidity, other airborne chemicals and radiation [110]. In order to manufacture artificial spider silk proteins, small-scale centrifugal electrospinning was used to create non-woven meshes for fine dust filters [111,112]. A comparison of artificially manufactured spider and lacewing silk fibres with polylactic acid (PLA) and polyethylene oxide (PEO) spun into non-woven filter media showed that silk-based media more efficiently retained smaller particles (0.1−1 µm) and yielded a lower pressure drop that improved air permeability [112]. Manufactured filter media mimicking the non-woven structure and material of hornet silk caps (§3.5) have not been tested yet. Insect silk could be a promising filter material as it also has hydrophobic properties that even prevent acids from permeating [113] and thermoelectric properties that have insulating effects [114,115].

In addition to silk, spiders use sticky drops to increase capture while increasing mesh size with less hydrodynamic drag (§3.6). Adhesive materials, like waxes on surface leaves (§3.1), mucus in nasal cavities (§3.2), or adhesive forces in pollen [116], are generally very common in biological PSM in air (table 2) to increase particle attachment. A bio-inspired attempt was made to mimic pollenkitt, which is responsible for the adhesion of pollen to pollinators, or the agglomeration of pollen themselves. Prototypes showed that the bio-inspired surface properties influence the adhesion of aerosol particles [117]. The sandfish lizard was used to design a prototype that uses the specific morphology and artificial mucus to increase particle adhesion. This avoids the use of a filter medium and associated pressure losses [55]. Adhesives are also common in aerosol filters. A simple example is sticky fly paper. Other designs include grease- or oil-covered impaction surfaces in impactors [102] or filter fibres in air conditioning filters [118]. Wetted-wall cyclones continuously flow liquid over the collection surface to remove collected particulate and concentrate it into the collection fluid [119], which appears like a similar concept to the continuous refreshing of the mucus in the MR region of the nasal cavity.

Most BMs, except phytoremediation and hornet silk caps, have an inherent cleaning mechanism. Cleaning is important because a functioning PSM needs to be maintained and a clogged separation medium can reduce filtration efficiency and increase aerodynamic drag [96]. Engineered cleaning solutions include brushing or scraping, reversed flow of compressed air, washing with fluids, vibration and ultrasonic or chemical cleaning [96,120]. Associated problems are the risk of damage, interrupted filtration processes, incomplete cleaning, increased operational costs and maintenance and release of harmful by-products [96,120]. Cleaning in BMs is done by ciliary and mucus transport in the nasal cavities, absorption through germination in plants, removal of captured prey particles as in spider webs, or airflow reversal in insect spiracles (table 2). The hornet silk caps are not cleaned, as they are simply destroyed when the adult hornets eat their way through it (§3.5). Therefore, silk caps resemble single-use, non-recyclable filters such as face masks [121]. By having an inherent cleaning mechanism, the other BMs can remain a continuous particle separation process (table 2). At the same time, materials involved in particle deposition are also used during the cleaning processes, e.g. mucus on the nasal cavities captures, accumulates and transports particles. This multi-functionality is often present in biological systems [36,122]. Multi-functionality is particularly apparent in the nasal cavities, which serve respiratory air conditioning and thermoregulation, olfaction and filtration of the air (§3.2). Multi-functionality often incorporates trade-offs. A trade-off in the biological context means that evolutionary processes do not lead to an optimized single function but instead increase the overall fitness of a population [123]. For example, in wind-pollination, plants have a high number of flowers to increase encounter rate but the flowers are small to increase resource efficiency (§3.3). In orb webs, the trade-off is made between a decrease of mesh size to capture more prey and an increase in mesh size to reduce aerodynamic drag and save material (§3.6). Multi-functionality and trade-offs are also found in man-made filters with high filtration efficiency versus low pressure drop being the most important one [96,124]. This review supports engineers and biologists in exploring a wider range of biological strategies for innovative filtration technologies. Biologists can perform comparative studies of BMs, either of closely related species or independent taxonomic groups, and aid in overcoming limitations of mimicking one specific trait in the biomimetic working process [26,36,125]. For example, when studying nasal cavities, it could be advantageous to investigate the turbinate regions of mammalian species that are adapted to temperate climates in comparison with species that are adapted to arid climates to identify respective trait changes for habitats with higher particle loads and different humidities. Engineers face challenges of scalability and transferability during the biomimetic working processes [126]. Scaling can have significant effects on the filtration performance. For example, an increase in filtration area can result in decreased flow resistance and increased volume flows, which affect flow characteristics, particle deposition and clogging [10,127].

In biology, scaling and its potential non-proportional relationships with shape, structure or function are analysed in allometry studies [128]. For example, studies of filter-feeding whales showed that the engulfment capacity, i.e. the filtered water volume, increases disproportionally with increased body length (positive allometry, slope = 3.3−3.8), which increases the energetic efficiency through prey capture but it also increases the energetic cost through additional drag [129]. In the nasal cavities of arctoids and canids foxes, bears and weasels, the turbinal surface area scaled proportionally with body size that ranged between 0.17 and 550 kg. However, the olfactory region showed disproportional scaling in canids, which indicates adaptions to hunt prey over large distances [130]. These adaptations to scaling may also be relevant in the transfer of BMs to technical applications. For example, upscaling of the feathery structures of flower stigmata is limited by the physical boundary conditions in which the PSM works, i.e. smaller fibre diameter decreases the boundary layer thickness and increases particle interception (§3.3). When transferring a biological design to a manufactured design, it may be advantageous to avoid scaling up the physical size and instead use multiple ‘units’ with the same sizes, e.g. repeating (decreasing or increasing the tortuosity) the turbinate structure in the nasal cavities in order to preserve the gap thickness to achieve laminar flow (§3.2). In filtration, the Reynolds number and the Stokes number are critical dimensionless numbers that should be maintained and show the limits of scaling a filtration process [10,127].

Other challenges regarding the transferability are finding man-made materials that exhibit the same properties as the biological materials. Biological materials can have complex stress–strain relationships, as in spider webs (§3.6), or complex fluid–structure interactions, as in the interaction of shape, surface roughness, mucus and airflow in the nasal cavities (§3.2). It might also be impractical or impossible to manufacture small features such as feathery structures or cilia. Advances in microfabrication, sensory systems and actuation technologies may enable the future transfer of small-scale biological features, such as cilia, into functional engineered systems. In studying BMs, parameters and principles that allow comparisons beyond homologous structures and taxonomic groups (table 2) can provide guidance in identifying biological models that fit engineering problems in separation technologies.

## Data Availability

Supplementary material is available online [131].
